# Clinical Application of an Oral Liquid Bandage (ORAPLA) for Traumatic and Surgical Oral Mucosal Wounds: A Technical Note

**DOI:** 10.3390/dj14020073

**Published:** 2026-02-02

**Authors:** Hiroshi Furuta, Atsushi Abe, Shoya Mizuno, Sayaka Furuhashi, Sayumi Hiraguri, Moeko Momokita, Tetsushi Oguma, Atsushi Nakayama, Hiroki Inoue

**Affiliations:** 1 Department of Oral Medicine and Oral Surgery, School of Dentistry, Aichi Gakuin University, Nagoya 464-8651, Japan; 2 Department of Oral and Maxillofacial Surgery, Shizuoka General Hospital, Shizuoka 420-0881, Japan; 3 Department of Oral and Maxillofacial Surgery, Daiyukai General Hospital, Ichinomiya 491-0036, Japan

**Keywords:** oral liquid bandage, oral mucosal wound, aphthous stomatitis, oral medicine

## Abstract

**Background/Objectives:** Oral mucosal wounds are frequently encountered in daily dental practice and are often difficult to manage because of continuous exposure to saliva, mastication, and mechanical irritation. This technical note describes the clinical practicality of an oral liquid bandage (ORAPLA) as a film-forming protective barrier for traumatic and surgical oral mucosal wounds. **Methods:** ORAPLA was applied in four clinical scenarios: a traumatic lip bite injury, a postoperative mucosal defect following leukoplakia excision, a biopsy wound for suspected oral squamous cell carcinoma (OSCC), and aphthous stomatitis. Clinical observations included patient-reported symptom relief, film retention, and the clinical appearance of epithelialization at follow-up (1–2 weeks). **Results:** In all cases, ORAPLA formed a thin protective film immediately after application and was typically observed to remain on the wound surface for approximately 5–6 h under routine daily activities. Patients reported prompt subjective pain relief, and no adverse events were observed. Epithelialization proceeded without clinically evident secondary infection during the follow-up period. **Conclusions:** In this small descriptive case series, ORAPLA was feasible to apply, well tolerated, and provided temporary mechanical protection with immediate subjective comfort. Controlled studies using standardized outcome measures are warranted.

## 1. Introduction

Oral mucosal wounds are frequently encountered in daily dental practice and arise from a wide range of etiologies, including accidental biting, irritation from ill-fitting dentures or orthodontic appliances, thermal or chemical trauma, surgical excision of potentially malignant disorders, and diagnostic biopsy of suspected malignant lesions [1]. Unlike cutaneous wounds, intraoral wounds exist in a uniquely challenging environment. The oral cavity is continuously exposed to saliva, temperature and pH fluctuations, a diverse and dense microbiome, and repeated mechanical stress during mastication, speech, swallowing, and oral hygiene. These factors can disrupt clot stability, increase frictional microtrauma, and complicate epithelial migration, thereby contributing to disproportionate symptoms and variable healing trajectories [1,2].

Even relatively small mucosal injuries may cause persistent pain, dysarthria, dysphagia, sleep disturbance, and reduced oral intake, negatively affecting quality of life and daily function. From a clinical standpoint, maintaining a stable wound surface and minimizing repeated irritation are key determinants for symptom control and uncomplicated epithelial regeneration. However, achieving such stability is inherently difficult in the oral cavity, where topical agents are readily diluted by saliva, displaced by the lips, cheeks, and tongue, or removed during eating and routine oral hygiene.

Among common oral mucosal disorders, recurrent aphthous stomatitis is one of the most prevalent inflammatory conditions and is characterized by episodic painful ulcerations with an unclear etiology. Immunologic dysregulation, genetic predisposition, microbial interactions, nutritional deficiencies, and psychological stress have been implicated [3]. Standard management strategies include topical corticosteroids, antiseptic mouth rinses, topical anesthetics/analgesics, and mucosal protectants [3,4]. Despite these interventions, many patients experience incomplete relief, partly because topical formulations frequently fail to remain localized on wet mucosa for adequate periods. In addition, concerns regarding repeated or prolonged use of pharmacologically active agents have increased interest in non-pharmacological supportive approaches that focus on mechanical protection and reducing frictional trauma.

The importance of physical protection and moisture maintenance is well established in wound management, where occlusive or semi-occlusive dressings, hydrogels, collagen matrices, and bioactive coverings are widely used to reduce contamination, preserve a favorable microenvironment, and mitigate mechanical irritation [1,5]. In contrast, comparable technologies adapted for the oral cavity remain limited. The dynamic mechanical and biochemical environment of the mouth renders conventional dressings impractical, and relatively few materials are specifically engineered for intraoral wound protection [5,6]. Therefore, a film-forming barrier that can adhere to wet mucosa and provide temporary mechanical protection may be clinically useful as an adjunct to routine care.

In addition to traumatic and postoperative wounds, many patients experience oral pain that is exacerbated by everyday mechanical irritation. This clinical reality underscores the potential utility of temporary, film-forming barriers that can adhere to wet mucosa and reduce frictional stimuli. While the present technical note does not evaluate disease-specific indications, it aims to describe a practical application protocol and clinical observations in representative wound scenarios. Accordingly, a ready-to-use oral liquid bandage designed to form a protective film on wet mucosa may be a pragmatic adjunct in routine practice.

ORAPLA (FUJIFILM Toyama Chemical Co., Ltd., Tokyo, Japan) was developed to address this unmet need. In Japan, the product is commercially available under a regulatory notification/registration framework pursuant to the Pharmaceuticals and Medical Devices Act; the cited identifier (notification number: 13B2X00129000001) serves as a regulatory listing/market distribution identifier and does not indicate intellectual property protection or novelty of the underlying technology. The formulation is protected by an international patent publication (WO2020/045133A1; WIPO, published on 5 March 2020). ORAPLA is intended to function as a non-pharmacological, mechanical barrier and does not contain pharmacologically active agents such as corticosteroids, local anesthetics, antibiotics, or antiseptics. In vitro studies have suggested that prolonged exposure of dental hard tissues to ORAPLA presents minimal risk of acid erosion, even after repeated contact [7]. Despite this profile, detailed clinical descriptions of performance across diverse oral wounds remain limited.

Accordingly, this technical note aims (i) to describe a reproducible clinical application protocol for ORAPLA and (ii) to report clinical observations regarding feasibility, short-term film retention, patient-reported comfort, and the clinical appearance of epithelialization in representative scenarios, including trauma, postoperative mucosal defects, biopsy wounds, and inflammatory ulceration.

## 2. Materials and Methods

### 2.1. Device Characteristics and Intended Function

ORAPLA is a semi-viscous, carbomer-based (carboxyvinyl polymer) oral liquid dressing designed to form a thin, transparent film on mucosal surfaces. Upon exposure to moisture (e.g., saliva), the polymer hydrates and forms a cohesive gel-like membrane through polymer chain entanglement and network formation. The resulting film is lightweight, flexible, and conformable, allowing it to adhere to wet mucosa without mechanical fixation. The device is intended to provide temporary mechanical protection by isolating exposed tissue from mechanical, thermal, and chemical stimuli during routine oral activity.

The formulation is supplied in a single-use pouch that allows hygienic extrusion of a controlled amount of material. This packaging reduces the risk of contamination and facilitates patient self-application outside clinical settings. In the present report, ORAPLA was applied using a cotton swab to achieve a thin, even layer over the target lesion.

### 2.2. Preclinical Safety Assessment

According to the manufacturer’s technical documentation, biocompatibility-related evaluations were performed in line with applicable regulatory frameworks (e.g., cytotoxicity, sensitization, and irritation testing). This technical note does not independently verify these tests; therefore, safety observations herein are limited to clinical follow-up within the included cases and the absence of clinically evident adverse reactions during the observation period.

### 2.3. Clinical Setting, Ethics, and Case Selection

Four representative cases were selected to illustrate feasibility across common clinical scenarios: (i) traumatic lip bite injury; (ii) postoperative mucosal defect following leukoplakia excision; (iii) biopsy wound for suspected oral squamous cell carcinoma; and (iv) aphthous stomatitis. All patients were treated at a university-based clinic specializing in oral medicine and oral surgery. Case descriptions were prepared with reference to the CARE guidelines for case reports. Written informed consent was obtained from all patients for treatment and for publication of anonymized clinical data and images. Ethics committee review was exempted according to our institutional policy for this descriptive technical note.

Because this is a descriptive technical note, no formal comparator, randomization, or standardized quantitative outcome instruments were applied. The purpose of case selection was to demonstrate practical application in heterogeneous, clinically relevant situations rather than to infer efficacy.

### 2.4. Standardized Application Protocol

**Figure 1:**
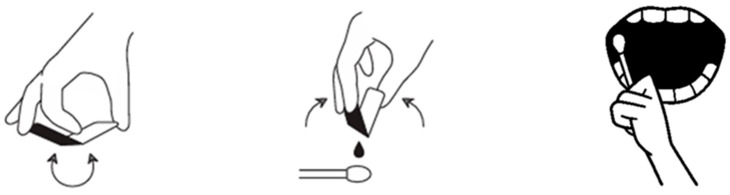
Application protocol: ORAPLA was applied in a thin layer using a cotton swab after gentle drying of the wound surface.

A standardized clinical application procedure was used across cases, with minor pragmatic adjustments based on lesion location and patient comfort. The workflow is summarized in Figure 1.

**1. Surface preparation (drying):** The lesion surface and immediate surrounding mucosa were gently dried using sterile gauze and/or an air syringe. Drying aimed to reduce excess saliva at the application site to improve initial adherence. If minor bleeding or exudate was present, gentle blotting was repeated until the surface appeared visibly less wet (without aggressive rubbing).

**2. Application method and layer thickness:** ORAPLA was extruded from the single-use pouch onto a cotton swab. A thin layer was applied directly over the lesion to cover the exposed surface. Excessive thickness was avoided to reduce premature displacement. Where feasible, a small margin of surrounding mucosa was lightly coated to help stabilize the film edge.

**3. Film formation:** Film formation was observed to occur within approximately 5–10 s after application. Successful film formation was confirmed clinically by the appearance of a continuous, glossy, transparent layer over the lesion surface.

**4. Post-application instructions:** Patients were instructed to avoid eating and drinking for at least 30 min after application. Additional counseling included avoiding deliberate peeling of the film, minimizing mechanical rubbing at the site, and avoiding highly irritating foods (e.g., spicy or strongly acidic foods) during the early period after application.

**5. Reapplication schedule:** ORAPLA was reapplied two to three times daily, preferably before meals, until subjective pain subsided. Patients were advised that reapplication could be performed earlier if the film was clearly displaced and discomfort recurred. No patient discontinued ORAPLA because of discomfort, adverse reactions, or perceived loss of adhesion.

### 2.5. Clinical Observation Items and Operational Definitions

Clinical observations focused on feasibility, patient-reported comfort, short-term film retention, and the clinical appearance of epithelialization.

✓ **Feasibility:** Feasibility was assessed by whether the material could be applied as intended (thin, continuous layer) and whether film formation was achieved promptly at the targeted intraoral site without immediate washout.
✓ **Patient-reported symptom relief:** Symptom relief was recorded as qualitative patient report (e.g., “improved,” “markedly improved,” or “no change”) within minutes after application and at follow-up. Validated pain scales were not used; therefore, these observations are descriptive.
✓ **Film retention:** Retention time was documented as clinical observation and/or patient report under routine daily activities (eating, drinking, speech, oral hygiene). Because objective instrumentation was not used, retention time should be interpreted as an estimated practical duration rather than a precise measurement.
✓ **Clinical appearance of epithelialization:** At follow-up visits (approximately 1–2 weeks, depending on case), the wound was visually assessed for reduction in erythema, coverage by intact epithelium, and absence of exposed connective tissue as clinically apparent indicators consistent with epithelialization.
✓ **Safety/tolerability:** Adverse events were assessed by patient report and clinical inspection, including unpleasant taste, burning sensation, foreign-body sensation, mucosal irritation/erythema beyond the lesion, suspected allergic reaction, and clinically evident signs of secondary infection (e.g., increasing swelling, purulent exudate, progressive erythema with worsening pain, or systemic symptoms).

### 2.6. Documentation

Standard clinical photographs were obtained at baseline and follow-up for each case, and additional photographs were taken immediately after application to document film formation where feasible. Images were anonymized prior to use in academic reporting. Because photographic conditions in routine practice may vary (lighting, angle, distance), images are presented as clinical documentation rather than quantitative measurement.

## 3. Results

### 3.1. Overall Clinical Findings

In all cases, ORAPLA formed a thin protective film immediately after application. Based on clinical observation, the film typically remained over the wound surface for approximately 5–6 h under routine daily activities, including eating, drinking, and oral hygiene. All patients reported subjective pain relief within minutes after application. No adverse reactions, allergic responses, taste disturbances, or clinically evident secondary infection were observed during follow-up.

### 3.2. Case Reports

**Case 1: Traumatic Lip Bite Injury**

**Figure 2:**
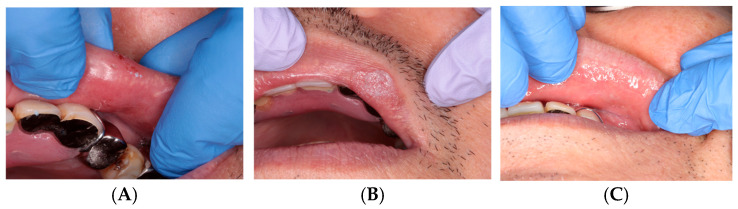
Traumatic bite injury: (**A**) Before treatment. (**B**) Immediately after ORAPLA application. (**C**) 1-week follow-up.

Film formation occurred immediately after application. The patient reported a progressive reduction in pain over several days, and the wound surface appeared epithelialized at the 1-week follow-up. The clinical appearance at follow-up is shown in Figure 2.

**Case 2: Postoperative Defect Following Leukoplakia Excision**

**Figure 3:**
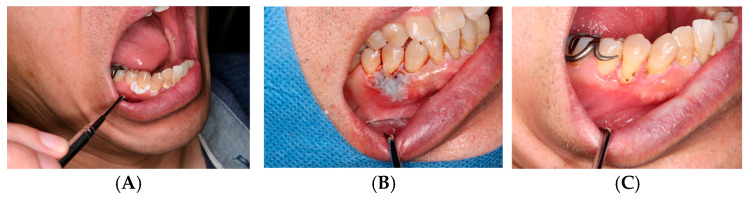
Leukoplakia excision: (**A**) Preoperative appearance. (**B**) ORAPLA applied postoperatively. (**C**) 2-week follow-up.

ORAPLA was used to provide temporary coverage of the postoperative mucosal defect. The wound appearance was consistent with progressive epithelialization at 2-week follow-up, without clinically evident secondary infection. See Figure 3.

**Case 3: Biopsy Wound for Suspected OSCC**

**Figure 4:**
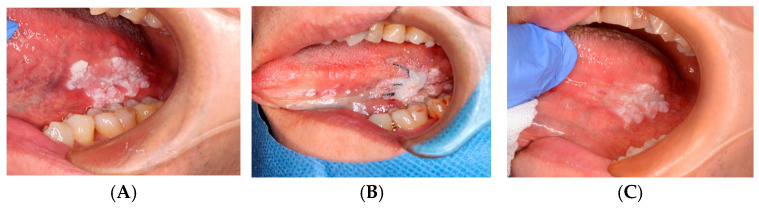
OSCC biopsy wound: (**A**) Pre-biopsy lesion. (**B**) ORAPLA applied. (**C**) 1-week follow-up.

ORAPLA was applied to the biopsy wound. The patient reported reduced discomfort, and the wound appearance was consistent with healing at the 1-week follow-up. See Figure 4.

**Case 4: Aphthous Stomatitis**

**Figure 5:**
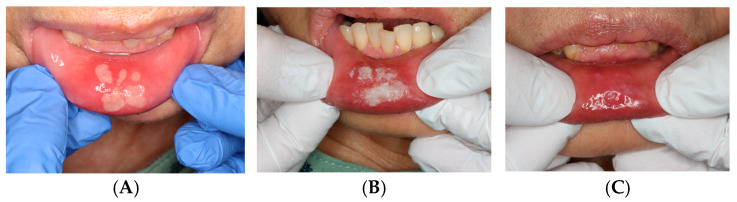
Aphthous stomatitis: (**A**) Initial presentation. (**B**) Immediately after the ORAPLA application. (**C**) 1-week follow-up.

ORAPLA was applied to painful aphthous ulcers. The patient reported prompt symptom relief, and the ulcers appeared clinically resolved at the 1-week follow-up. See Figure 5.

## 4. Discussion

This technical note describes clinical observations regarding the practicality of ORAPLA as a film-forming oral liquid bandage across several representative oral mucosal wound scenarios (trauma, postoperative defect, biopsy wound, and aphthous ulceration). The primary observations were immediate film formation, temporary retention under intraoral conditions, and patient-reported subjective comfort during the early healing period, without adverse events in this small series.

### 4.1. Role of Mechanical Protection in Oral Wound Management

A central challenge in oral wound management is maintaining a stable wound environment. Intraoral lesions are continuously exposed to saliva, temperature changes, a dense microbiome, and repeated mechanical stress from mastication, swallowing, and speech. These factors can contribute to ongoing irritation and pain and may interfere with wound stabilization [1,2]. In principle, barrier-based approaches can reduce mechanical irritation and help maintain a moist microenvironment, which is favorable for epithelial migration. However, developing intraoral barriers is challenging because many topical agents are easily displaced or diluted. ORAPLA is intended to address this challenge by forming a thin, flexible film directly on wet mucosa. In the present cases, the film was typically observed to remain for approximately 5–6 h under routine daily activities. This transient but meaningful retention may be sufficient to reduce repeated irritation during eating and speaking and may explain the immediate subjective comfort reported by patients.

### 4.2. Context Within Existing Intraoral Wound-Care Options

Several intraoral protective approaches are used in practice, including bioadhesive pastes, oral patches, and barrier materials used after oral surgical procedures [5,6]. Each has practical limitations in the oral environment related to placement, dilution by saliva, or short contact time. ORAPLA differs in that it is designed primarily as a mechanical barrier that self-forms into a film after application. Importantly, the present report is an uncontrolled descriptive case series; therefore, no comparative claims can be made regarding efficacy versus other products or standard care. The observations herein should be interpreted as hypothesis-generating and intended to inform future controlled evaluations.

### 4.3. Safety and Tolerability

In this case series, no adverse events were observed, and patients did not report unpleasant taste, burning sensation, or mucosal irritation. However, given the small sample size and limited follow-up, broad safety conclusions—especially for specific populations—cannot be drawn from the current data. In vitro findings suggesting minimal erosive potential on dental hard tissues provide a supportive context for incidental contact with dentition during intraoral use [7].

### 4.4. Limitations

Several limitations must be acknowledged. First, the sample size is small, and the design is descriptive, with no control or comparator. Second, outcome measures were primarily subjective, and no validated pain scales or quantitative wound assessments were employed. Third, retention time was not quantified using objective measurement methods and was recorded as a clinical observation. Finally, follow-up intervals varied slightly across cases. Accordingly, the present findings should be regarded as preliminary and hypothesis-generating.

### 4.5. Future Perspectives

Future prospective studies are warranted to define where film-forming barrier therapy may offer a clinically meaningful benefit, beyond the heterogeneous scenarios described in this technical note. Because ORAPLA is designed as a non-pharmacological, mechanical barrier, its potential value may be greatest in conditions in which symptoms are driven by repeated mechanical/chemical irritation of exposed epithelium and in which conventional topical formulations are limited by dilution and displacement in the oral cavity. Importantly, the following perspectives are speculative and hypothesis-generating; they are not directly evaluated in the present four-case series.

#### 4.5.1. Immune-Mediated Oral Mucosal Disease

Several immune-mediated disorders are characterized by epithelial fragility, recurrent erosions/ulcerations, and disproportionate pain relative to visible mucosal changes. Oral lichen planus (particularly erosive/ulcerative phenotypes) is a common example associated with chronic burning pain and impaired oral function [8]. Likewise, graft-versus-host disease after hematopoietic stem cell transplantation may involve mucosal atrophy, ulceration, salivary dysfunction, and heightened susceptibility to secondary trauma [9]. In these settings, routine oral activities (speech, mastication, oral hygiene) can repeatedly disrupt fragile epithelium, amplifying nociceptive input and perpetuating symptoms. A film-forming mechanical barrier could be explored as an adjunct supportive approach aimed at reducing microtrauma and shielding exposed nerve endings from external stimuli. Future studies should define patient selection, assess tolerability on inflamed mucosa, and incorporate validated symptom endpoints.

#### 4.5.2. Relevance to Oncology Supportive Care and Oral Mucositis

Oral mucositis remains a major dose-limiting toxicity of cancer therapy, with substantial impact on pain, nutrition, oral intake, and quality of life [10]. Current management is largely supportive, and durable physical protection of ulcerated mucosa is difficult to achieve in the oral environment [10,11]. Given its mechanical mode of action and the absence of pharmacologically active ingredients, a film-forming barrier may be conceptually compatible with supportive care needs in medically complex patients, including those in whom drug–drug interactions, systemic absorption, or mucosal irritation are concerns. Prospective trials should incorporate standardized mucositis grading, validated pain scores, objective documentation of film retention, and patient-centered outcomes such as the ability to eat and speak.

#### 4.5.3. Toward Drug-Embedded Film Technology

Although ORAPLA is currently positioned as a non-pharmacological barrier, film-forming platforms may be adaptable for localized drug delivery in the future. Incorporation of anti-inflammatory agents, analgesics, antimicrobials, or growth-modulating compounds could enable dual-function systems that provide both mechanical protection and sustained local exposure with minimal systemic absorption. Such development would require rigorous formulation and regulatory evaluation, including stability, release kinetics, mucosal permeability, and irritation potential in the dynamic intraoral environment [5].

From a translational perspective, key engineering targets include controllable film thickness and dissolution time, optimization of adhesion under saliva flow, and predictable retention in high-mobility anatomical sites. The oral cavity poses unique challenges compared with cutaneous drug delivery (continuous moisture, mechanical shear, enzymatic activity, and dense microbiome). Accordingly, future research should proceed stepwise, beginning with in vitro characterization and ex vivo mucosal models, followed by carefully designed early-phase clinical studies.

#### 4.5.4. Study Design Considerations for Future Trials

To establish evidence-based guidance, future clinical studies should incorporate standardized and reproducible outcome measures: (i) validated pain scales (e.g., numeric rating scale or VAS) recorded at predefined timepoints; (ii) objective or semi-objective assessment of film retention time (e.g., patient diary with structured timepoints, photographic documentation, or clinician verification); (iii) quantitative wound assessment where feasible (lesion dimensions, epithelialization score, or standardized photographic analysis); and (iv) safety endpoints including irritation, taste disturbance, and signs of secondary infection. Comparative designs (e.g., standard care alone vs. standard care plus barrier therapy) would be particularly informative for defining incremental benefit and identifying indications where barrier formation translates into meaningful patient-centered improvement.

## 5. Conclusions

In this small descriptive case series, ORAPLA was feasible to apply, well tolerated, and provided temporary mechanical protection with immediate subjective comfort in several representative oral mucosal wound scenarios. Given the practical challenges of maintaining topical coverage in the oral cavity, film-forming barrier therapy merits further evaluation in indication-specific prospective studies, including settings characterized by epithelial fragility or treatment-related mucosal injury.

## Data Availability

The data presented in this study are not publicly available due to patient privacy and ethical restrictions. Anonymized data may be made available from the corresponding author upon reasonable request.
